# Evaluation of the Antioxidant, Anticancer, and Antibacterial Activities of Flower and Leaf Extracts of *Chrysanthemum* *indicum*

**DOI:** 10.1002/open.202500252

**Published:** 2025-06-05

**Authors:** Tarad Abalkhail, Noorah A. Alkubaisi, Ibrahim M. Aziz, Mashail Fahad Alsayed, Hissah Abdulrahman Alodaini, Amal Saad AL‐Shenifi, Mohamed A. Farrag

**Affiliations:** ^1^ Department of Botany and Microbiology College of Science King Saud University Riyadh 11451 Saudi Arabia

**Keywords:** antibacterial activities, anticancer potentials, antioxidant activities, natural products, suppressive effects

## Abstract

Chrysanthemum has been studied for its anti‐inflammatory, antibacterial, anticancer, antioxidant, and other pharmacological properties. However, there is little knowledge about the methanol–pharmaceutical activities of *Chrysanthemum indicum* leaves and flowers. This study is designed to assess the in vitro antioxidant, anticancer, and antibacterial activities of *C. indicum* extracts. The flowers and leaves of *C. indicum* are extracted. Gas chromatography–mass spectrometry is used to analyze the chemical components. The antioxidant activity is evaluated using 2,2‐diphenyl‐1‐picrilhidrazine (DPPH) and 2,2′‐azino‐bis (3‐ethylbenzothiazoline‐6‐sulfonic acid) (ABTS) assays. Cytotoxic effect against A549 cell line is identified. The antibacterial properties are evaluated against both Gram‐positive and Gram‐negative bacteria. Antioxidant activity is detected and observed to be maximum at higher concentrations. The IC50 values of flowers and leaves are 77.19 ± 2.4 and 101.94 ± 2.34 μg mL^−1^, respectively, in the DPPH method, whereas the ABTS assay shows 93.21 ± 3.42 and 98.22 ± 3.34 μg mL^−1^, respectively. The flower extract has more cytotoxic activity than the leaves (*P* < 0.05). The IC50 values for flowers and leaves are 72.49 ± 3.14 and 102.54 ± 4.17 μg mL^−1^, respectively. Furthermore, the flower and leaf extracts exhibit the most pronounced antibacterial activity. These results provide a strong basis for further research into its potential therapeutic uses as well as opportunities for the creation of natural pharmaceutical products.

## Graphical abstract

The tested methanolic extract of *Chrysanthemum indicum* flower and leaf is a rich source of bioactive molecules with significant antioxidant activity at low doses, potential anticancer effects at lower concentrations, and antimicrobial activities against Gram‐positive and Gram‐negative species, which is consistent with the plant's traditional applications.

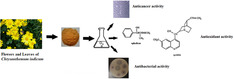

## 1 Introduction

Medicinal plants have a wide range of bioactive secondary metabolites that may be utilized to identify and develop novel chemical compounds against pathogenic organisms. About 50% of cancer drugs have been isolated from plant sources. The therapeutic value of medicinal plants varied based on the presence of phytochemicals such as alkaloids, flavonoids, phenolics, and terpenes.^[^ ^1^ ^]^ Flavonoids are the major class of phytochemical compounds that show maximum antioxidant properties, and their consistent consumption would be useful to prevent the development of cancer.^[^ ^2^ ^]^

The genus *Chrysanthemum,* also called mums, is a small *Artemisiinae* genus, which is native to northern Europe and East Asia and has around 40 species, as well as several hybrids and cultivars. It is often used in several nations, such as China and Thailand, to prepare herbal tea.^[^ ^3^ ^]^ Numerous *Chrysanthemum* species of this genus have been recognized for their beneficial environmental impacts.^[^ ^4^ ^]^ *C. morifolium* flower pods were used for the preparation of tea drinks.^[^ ^5^ ^]^ In China, *C. morifolium* is popular and has been widely used to improve eyesight and to treat headaches, common colds, painful eyes, dizziness, and inflammation.^[^ ^6^ ^]^ In *C. morifolium*, the presence of polysaccharides, flavonoids, volatile oils, trace elements, and chlorogenic acid was reported. Flavonoids extracted from *C. morifolium* improved antioxidant activity and alleviated lead poisoning, antagonized oxidative injury of the liver, brain, and kidney, and reduced lipid peroxidation reaction.^[^ ^7^ ^]^

*C. indicum* was reported in various countries, including Thailand, China, and East Asian Countries.^[^ ^3^ ^]^ The whole plant was used for the preparation of pharmaceutical agents. Moreover, the flower has been used for 2000 years in Oriental countries, including China, Korea, Japan, and Vietnam, to prepare *chrysanthemum* tea.^[^ ^4^ ^]^ *C. indicum* is used to treat colitis, diarrhea, stomatitis, soreness, fatigue, pertussis, hypertensive symptoms, and vertigo. The phytochemicals, such as glycosides, flavonoids, and phenols, were determined from *C. indicum*. These phytochemicals presented antibacterial activity against various bacterial and fungal pathogens.^[^ ^8^ ^]^

Remarkably, the cytotoxic and antibacterial qualities of the *leaves and flower* extracts have only been partially elucidated by a small number of preliminary investigations in the field of *C. indicum* research. The antibacterial, antioxidant, and anticancer effects of extracts from *leaves and flowers*, however, are not well studied. *C. indicum* was able to significantly inhibit the cell cycle by regulating cell cycle–related proteins in MHCC97H cells and cause apoptosis through a mitochondrial route when extracted in 95% ethanol. This effect was not observed in normal cells.^[^ ^9^ ^]^ Furthermore, at nontoxic quantities, treatment with *C. indicum* extracts markedly sensitized the multidrug‐resistant human breast cancer cell line MCF7/ADR. Significant antioxidant activity was shown by the ethanolic floral extract, as well as the aqueous and n‐hexane fractions of *C. indicum* in the 2,2‐diphenyl‐1‐picrilhidrazine (DPPH) technique with IC_50_ values of 1.350, 1.109, and 7.588 μg mL^−1^, respectively.^[^ ^10^ ^]^ The flower heads of *C. indicum* essential oil demonstrated a moderate level of antibacterial activity against the Gram‐positive bacteria that were tested, with a minimum inhibitory concentration (MIC) of 62.5 μg mL^−1^ for *Bacillus subtilis*, *Streptococcus agalactiae*, and *Streptococcus pyogenes*. However, weak activity against the tested strains of fungi and Gram‐negative bacteria with MICs greater than 500 μg mL^−1^.^[^ ^11^ ^]^ By carefully analyzing methanol extracts made from *Chrysanthemum indicum's* leaves and flowers, our study seeks to close this gap in the literature and advance knowledge in the field.

However, there is still a noticeable lack of research on the antioxidant, anticancer, and antibacterial properties of the flowers and leaves of *C. indicum*. Although a lot of material has been written on an extract of *C. indicum*, our study closes a big gap since this plant has not been well studied scientifically, despite its well‐known traditional therapeutic use. However, no comparison of the antioxidant, anticancer, and antibacterial properties of methanol extracts of *C. indicum* flowers and leaves has been performed. The objective of the present investigation was to conduct a comprehensive assessment of the chemical characteristics and the antioxidant, anticancer, and antimicrobial effects of extracts from the flowers and leaves of *C. indicum*. A unique technique that exposes a unique and intricate chemical composition is a thorough chemical analysis using gas chromatography–mass spectrometry (GC–MS). This study quantifies the amounts of total phenolic contents (TPCs) and total flavonoid content (TFC) and offers compelling proof of the extract's potent antioxidant, anticancer, and antibacterial effects. Furthermore, this work further elucidates the underlying processes that determine the anticancer of *C. indicum* flowers and leaves extracts by employing quantitative reverse‐transcriptase polymerase chain reaction (PCR) (RT‐qPCR)‐based mRNA expression profiling of particular pro‐ and antiapoptosis marker genes.

## 2 Results

### 2.1 Chemical Composition

**Figure 1:**
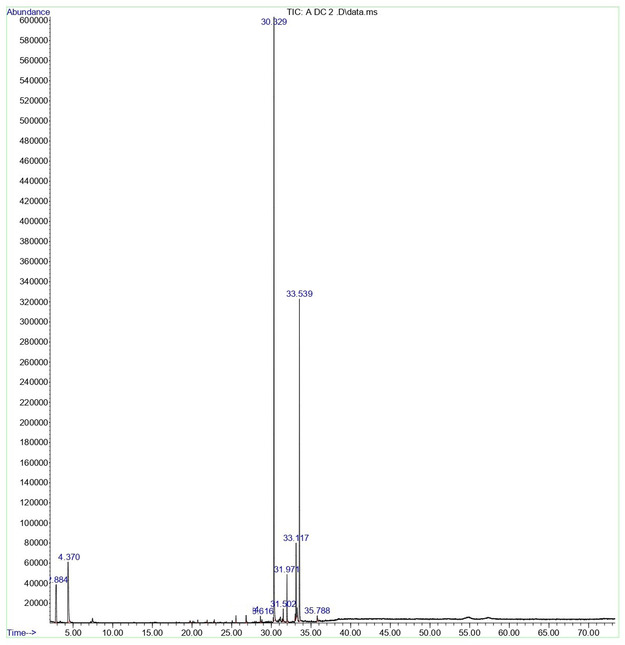
GC–MS spectra of secondary metabolites extracted in methanol from *C. indicum* flowers. A 70 min schedule was set, and about 1 μL of extract was administered.

**Figure 2:**
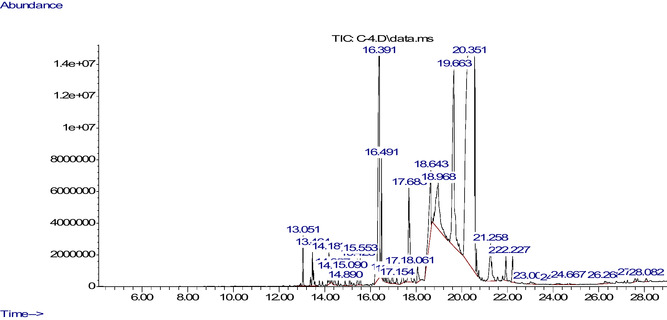
GC–MS spectrum of a methanol extract derived from the leaves of *C. indicum*. The GC–MS apparatus was set up for 70 min, and the methanol extract was used for the analysis. After injecting around 1 μL of extract, the device was set for 70 min. Sixteen compounds in all were identified.

The chemicals from the flowers and leaves of *C. indicum* were identified using GC–MS analysis. The main component in the flower's methanol extract was found to be hexadecanoic acid, methyl ester (43.86%), which was followed by methyl stearate (23.51%) and 2‐pentanone, 4‐hydroxy‐4‐methyl‐ (11.75%) (**Table** **1**; **Figure** **1**). And, 2‐cyclohexen‐1‐one, 2‐(2‐methyl‐2‐propenyl)‐69.75%) was the main component found in the leaf extract; it was followed by 9‐dodecenoic acid, methyl ester, (E)‐(14.03%) (**Table** **2** and **Figure** **2**).

**Table 1:** GC–MS identified volatile components from the methanol extract of *C. indicum* flowers.

No.	Hit name	Retention time [min]	Area	Area [%]
1.	2‐Pentanone, 4‐hydroxy‐4‐methyl‐	2.884	4,102,460	11.75
2.	Propane, 2,2‐diethoxy‐	4.37	2,627,046	7.524
3.	β‐Sitosterol	19.753	67,920	0.19
4.	Methyl 3‐bromo‐1‐adamantaneacetate	20.726	55,934	0.160
5.	11,12‐Dihydroxyseychellane	21.907	66,012	0.189
6.	Naphthalene, 1,2,3,5,6,8a‐hexahydro‐4,7‐dimethyl‐1‐(1‐methylethyl)‐, (1S‐cis)‐	22.807	50,273	0.143
7.	2‐(4a,8‐Dimethyl‐2,3,4,5,6,8a‐hexahydro‐1H‐naphthalen‐2‐yl)propan‐2‐ol	25.539	163,179	0.467
8.	Tridecanoic acid, methyl ester	26.814	275,307	0.788
9.	Decanoic acid, 2‐methyl‐	28.616	169,582	0.48
10.	Hexadecanoic acid, methyl ester	30.329	15,314,951	43.86
11.	Hexadecanoic acid, 15‐methyl‐, methyl ester	31.502	274,452	0.78
12.	Heptadecanoic acid, methyl ester	31.971	1,213,722	3.47
13.	9‐Octadecenoic acid (Z)‐, methyl ester	33.117	2,164,308	6.199
14.	Methyl stearate	33.539	8,207,071	23.51
15.	9‐Dodecenoic acid, methyl ester, (E)‐	35.788	160,982	0.46

**Table 2:** GC–MS identified volatile components from the methanol extract of *C. indicum* leaves.

No.	Hit name	Retention time [min]	Area	Area [%]
1.	Cyclohexane, 1‐ethenyl‐1‐methyl‐2,4‐bis(1‐methylethenyl)‐	13.05	6,144,141	1.21
2.	Benzene, 1‐(1,5‐dimethyl‐4‐hexenyl)‐4‐methyl‐	14.18	4,843,799	0.96
3.	Methyl 3‐bromo‐1‐adamantaneacetate	14.42	2,205,949	0.43
4.	*cis*‐α‐Bisabolene	14.89	871,176	0.17
5.	Caryophyllene oxide	15.42	3,302,761	0.65
6.	11,12‐Dihydroxyseychellane	15.55	3,706,309	0.73
7.	Z‐α‐*trans*‐Bergamotol	16.95	1,721,787	0.341
8.	9‐Octadecenoic acid (Z)‐, methyl ester	18.96	3,994,708	0.79
9.	(3H)‐Benzofuranone, 6‐ethenylhexahydro‐6‐methyl‐3‐methylene‐7‐(1‐methylethenyl)‐, [3aS‐(3a α, 6α, 7β, 7aβ)]‐	18.96	34,085,664	**6.76**
10.	9‐Dodecenoic acid, methyl ester, (E)‐	19.66	70,768,161	**14.03**
11.	2‐Cyclohexen‐1‐one, 2‐(2‐methyl‐2‐propenyl)‐	20.35	351,687,843	**69.75**
12.	9,12‐Octadecadienoic acid (Z,Z)‐	21.25	15,623,187	3.09
13.	Diazoprogesterone	23.00	1,426,021	0.28
14.	Hexadecanoic acid, 2‐hydroxy‐1‐(hydroxymethyl)ethyl ester	24.66	1,220,154	0.24
15.	n‐Propyl 9,12‐octadecadienoate	26.26	1,624,802	0.32
16.	Bicyclo[10.1.0]tridec‐1‐ene	28.08	916,774	0.18

### 2.2 TPC and TFC Analysis

The amount of TPC and TFC varied significantly (*P *< 0.05) between flowers and leaves of *C. indicum.* The recorded results revealed the potential of methanol as an effective organic solvent for the extraction of phenols and flavonoids. The methanol extract of flowers *C. indicum* presented the maximum amount of TPC and TFC were found to be 210.24 ±  3.43 and 95.24 ±  2.32 mg QE g^−1^ (quercetin equivalent [QE]) dry weight of the dry extract, which were significantly higher than the methanolic extract of leaves, 125.24 ±  4.41 and 73.22 ±  3.34 mg QE g^−1^, which contained dry weight of the dry extract, respectively.

### 2.3 Antioxidant Activity

**Figure 3:**
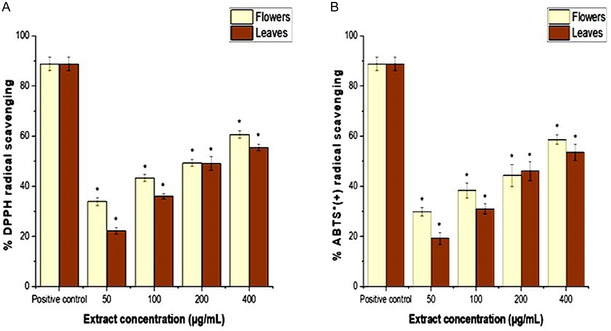
Antioxidant activity of flowers and leaves of *C. indicum* extract: A) DPPH radical scavenging activity and B) ABTS radical scavenging activity at various concentrations. Vitamin C (250 μg mL^−1^) was used as a positive control. The findings were presented as the average ± SD of three different experiments. The scavenging activity of the flower and leaf extracts was significantly lower (*) than the positive control at a significance level of *P *< 0.05. ^•+ ^=radical cation.

The antioxidant capacity of the phytochemicals from the flowers and leaves of *C. indicum* was analyzed using two different methods. DPPH and 2,2′‐azino‐bis (3‐ethylbenzothiazoline‐6‐sulfonic acid) (ABTS) radical scavenging activity results are described in **Figure** **3**. The results were compared with standard antioxidants (vitamin C, 250 μg mL^−1^). At increased extract concentrations, antioxidant activities were improved in both the ABTS and DPPH methods. The IC_50_ values of flowers and leaves were 77.19 ± 2.4 and 101.94 ± 2.34 μg mL^−1^, respectively, in the DPPH method, whereas the ABTS assay showed 93.21 ± 3.42 and 98.22 ± 3.34 μg mL^−1^.

### 2.4 Anticancer Activity

**Figure 4:**
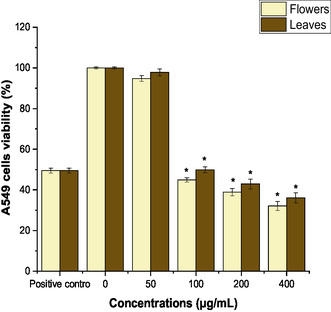
Cytotoxic activity of flowers and leaves extract of *C. indicum* at different concentrations (0–400 μg mL^−1^) and positive control (cisplatin 30 μg mL^−1^) after 24 h treatment. The findings were presented as the average ± SD of three different experiments. At higher concentrations, flower and leaf extract significantly decreased (*) cell viability (*P* < 0.05) compared to the negative control.

The cytotoxicity activity of the flowers and leaves of *C. indicum* extracts was analyzed. Remarkably, compared to the positive control (cisplatin 30 μg mL^−1^), the flowers and leaves of *C. indicum* extracts demonstrated notable anticancer potential against A549, in a concentration‐dependent manner (**Figure** **4**). The flower extract showed more potent cytotoxic activity than the leaves and was significant (*P* < 0.05). After 100 μg mL^−1^ extract in the culture medium, cytotoxic activity was less. However, increasing concentrations showed improved cytotoxic activity. The IC_50_ values for flowers and leaves were 72.49 ± 3.14 and 102.54 ± 4.17μg mL^−1^, respectively.

### 2.5 Analysis of Apoptosis and Antiapoptosis Genes

**Figure 5:**
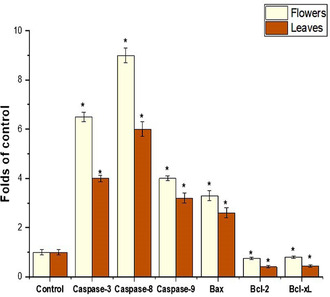
Effect of flowers and leaves extract of *C. indicum* extract on A549 cell and analysis of pro‐ and genes *caspase‐3, 8* and 9, and *Ba),* and antiapoptosis markers *(Bcl‐2* and *Bcl‐Xl*) genes. The findings were presented as the average ± SD of three different experiments. Gene expression was significantly (*) (*P* < 0.05) different from the negative control.

The effect of flower and leaf extract on A549‐induced apoptosis signaling was evaluated using RT‐qPCR after 24 h treatment. As described in **Figure** **5**, the flower and leaf extracts of *C. indicum* increased the expression of all three caspase mRNA levels. The result was compared with the untreated control, and flower extract treated with A549 after 24 h treatment showed an increased level of *Bax* mRNA and subsequently reduced antiapoptotic genes (*Bcl‐xL* and *Bcl‐2*) than the control (*P* < 0.05).

### 2.6 Antibacterial Activity

The antibacterial activity experiment demonstrated that both the flowers and leaf extracts of *C. indicum* displayed inhibitory effects on a range of investigated microorganisms. The findings, presented in **Table** **3** and **4**, demonstrate the antimicrobial efficacy of flower and leaf extract of *C. indicum* against several Gram‐positive and Gram‐negative pathogenic bacteria and were contrasted with conventional positive control (ciprofloxacin, 25 μg mL^−1^). Both extracts exhibited notable inhibitory zones against the tested bacteria gradually as concentration increased; at 500 and 1,000 μg mL^−1^, the inhibitory zone started to rise significantly (*P* < 0.05), although not as much as the positive control (25 μg mL^−1^ of ciprofloxacin). The flower extract showed more antibacterial activity, MIC 7.81–31.25 μg mL^−1^ (Table 3), than the leaves (MIC = from 15.62 ± 3.22 to 125 ± 3.28 μg mL^−1^).

**Table 3:** The inhibitory zone (mm), MIC (μg mL^−1^), and minimum bactericidal concentration (MBC) (μg mL^−1^) of *C. indicum* flowers extract.

Bacterium/dilution	Positive control	1,000 μg mL^−1^	500 μg mL^−1^	250 μg mL^−1^	125 μg mL^−1^	MIC μg mL^−1^	MBC μg mL^−1^
*Staphylococcus aureus* (MTCC 29213)	24 ± 1.61	21 ± 2.61a)	19.5 ± 2.93a)	14.5 ± 0.37a)	9 ± 0.29a)	15.62 ± 3.29	31.25 ± 2.98
*Staphylococcus epidermidis* (MTCC 12228)	39 ± 1.13	31 ± 1.91a)	26 ± 1.33a)	19 ± 1.93a)	14 ± 1.76a)	7.81 ± 1.53	15.62 ± 1.77
*Bacillus subtilis* (MTCC 10400)	25 ± 1.46	21 ± 1.33a)	19 ± 1.92a)	15 ± 1.19a)	11.5 ± 1.42a)	15.62 ± 1.37	31.25 ± 1.45
*Escherichia coli* (ATCC 25922)	25 ± 2.17	17 ± 2.53a)	16 ± 1.22a)	11.5 ± 1.44a)	10.4 ± 3.31a)	31.25 ± 1.38	62.5 ± 1.54
*Klebsiella pneumoniae* (MTCC 13883)	27 ± 1.32	16.6 ± 2.42a)	14 ± 2.89a)	11 ± 1.36a)	9.8 ± 2.14a)	31.25 ± 2.45	62.5 ± 3.44
*Pseudomonas aeruginosa* (MTCC 27853)	27 ± 2.25	19 ± 2.35a)	18 ± 1.26a)	15 ± 1.26a)	11 ± 2.24a)	15.62 ± 2.33	31.25 ± 2.43

a) In the same column, values that are significantly different from the positive control were determined by Student's *t* test, with *P* < 0.05 considered significant.

**Table 4:** The inhibitory zone (mm), MIC (μg mL^−1^), and MBC (μg mL^−1^) of the *C. indicum* leaves extract.

Bacterium/dilution	Positive control	1,000 μg mL^−1^	500 μg mL^−1^	250 μg mL^−1^	125 μg mL^−1^	MIC μg mL^−1^	MBC μg mL^−1^
*S. aureus* (MTCC 29213)	24 ± 1.61	17 ± 1.67a)	15 ± 2.33a)	12 ± 2.38a)	8.5 ± 3.15a)	31.25 ± 2.47	62.5 ± 3.23
*S. epidermidis* (MTCC 12228)	39 ± 1.13	25 ± 3.41a)	20 ± 2.11a)	15 ± 1.26a)	10 ± 1.71a)	15.62 ± 1.37	31.25 ± 1.45
*B. subtilis* (MTCC 10400)	25 ± 1.46	20 ± 3.32a)	16 ± 2.52a)	11 ± 3.74a)	9.6 ± 3.01a)	31.25 ± 4.42	62.5 ± 2.29
*E. coli* (ATCC 25922)	25 ± 2.17	16 ± 2.52a)	14 ± 1.13a)	9 ± 1.65a)	7 ± 1.34a)	125 ± 3.28	250 ± 3.28
*K. pneumoniae* (MTCC 13883)	27 ± 1.32	18 ± 2.52a)	15 ± 1.13a)	13 ± 1.36a)	9 ± 1.36a)	31.25 ± 356	62.5 ± 2.78
*P. aeruginosa* (MTCC 27853)	27 ± 2.25	16 ± 1.51a)	14 ± 1.33a)	10 ± 2.36a)	7 ± 2.19a)	15.62 ± 3.22	31.25 ± 4.57

a) In the same column, values that are significantly different from the positive control were determined by Student's *t* test with *P* < 0.05 considered significant.

## 3 Discussion

Several drugs based on plant‐derived compounds are being used in pharmaceuticals, and they have been used for centuries for a variety of purposes.^[^ ^12^ ^]^ Plant natural products comprising alkaloids, polyphenols, terpenoids, and flavonoids have been shown to have anticancer, antioxidant, anti‐inflammatory, and antibacterial properties.^[^ ^13^, ^14^ ^]^

Several chemical components of *C. indicum* have been studied before, with 36.696% representing the main compound, followed by isoborneol (7.64%), α‐terpinene (5.73%), and caryophyllene oxide (5.46%).^[^ ^11^ ^]^ GC–MS was used in our investigation to analyze a variety of phytochemical compounds. The main photochemical component in the obtained methanol extract of flowers was found to be hexadecanoic acid, methyl ester (43.86%), while the major photochemical component in the obtained methanol extract of leaves was found to be 2‐cyclohexen‐1‐one, 2‐(2‐methyl‐2‐propenyl) (69.75%). The hexadecanoic acid, methyl ester, has antibacterial, antioxidant, antiatherosclerotic, and anticancer properties.^[^ ^15^, ^16^ ^]^

In this study, the amount of TPC and TFC was determined from the leaves and flowers of *C. indicum.* The amount of TPC and TFC varied significantly (*P* < 0.05) between flowers and leaves of *C. indicum extract.* The methanol extract of flowers of *C. indicum* presented the maximum amount of TPC and TFC was found to be 210.24 ±  3.43 and 95.24 ±  2.32 mg QE g^−1^ dry weight of the dry extract, higher than those observed in the leaves. Similarly, the TPCs of 296.89 ± 14.53 mg gallic acid equivalent (GAE) g^−1^ extract dry weight (DW) and the TFCs of 361.03 ± 20.18 mg QE g^−1^ extract DW were shown by the ethanol extract of *Pancratium maritimum* seeds. In contrast, the flower extract had a TFC of 272.12 ± 16.42 mg QE g^−1^ extract DW and a TPC of 95.03 ± 7.22 mg GAE g^−1^ extract DW. Consequently, the phenolic and flavonoid levels of the ethanol extract of *P. maritimum* seeds are higher than those of the flower extract.^[^ ^17^ ^]^ The flower of *Chrysanthemum sp. exhibited* flavonoids, phenolic acids, and lignans with potential biological properties.^[^ ^18^ ^]^ Wu et al. (2010) reported the presence of myricetin and quercitrin in *C. indicum* flowers. The amount of phenolic content varied widely among the cultivars. The phenolic content varied from 74.0 to 123.7 mg GAE g^−1^.^[^ ^19^ ^]^ The amount of phenolic content determined in this study was higher than the report of Gong et al.^[^ ^20^ ^]^ Polyphenolic compounds and flavonoids provide health benefits. The amount of polyphenolic content varied based on the cultivar and states of the flower, and >47 mg g^−1^ fresh weight was reported.^[^ ^21^ ^]^

In our study, the methanolic extract exhibited potent antioxidant activity and was concentration dependent. The IC_50_ value obtained in our study in the DPPH method was lower than in previous reports.^[^ ^22^ ^]^ The methanolic extract of the flower extract showed the presence of chlorogenic acid, caffeic acid, and quercetin. The antioxidant activity of the methanolic extract of *C. indicum* might be due to the presence of these bioactive compounds.^[^ ^23^ ^]^ *C. morifolium* extract showed antioxidant activity, and the antioxidant capacity was determined using Cupric Reducing Antioxidant Capacity.^[^ ^24^ ^]^ A recent study also reported that the analysis of the leaves and flowers of several varieties of *C. morifolium* revealed that the leaves as well as the flowers contained significant amounts of phenolic compounds and showed potent antioxidant properties, as determined by the DPPH and ABTS assays.^[^ ^25^ ^]^ Moreover, the antioxidant capacity was lower than in the present study.

Cancer is a common disease that shortens people's lives. Cancer chemoprevention has been advocated as a viable method for lowering cancer incidence and mortality since 1970.^[^ ^26^ ^]^ Natural compounds may be beneficial in cancer treatment, mostly as chemopreventive agents.^[^ ^27^ ^]^ The search for new compounds with anticancer characteristics is continuously progressing.^[^ ^28^ ^]^ The limits of current cancer drugs have prompted anticancer pharmaceutical researchers to seek novel approaches to cancer treatment. For the past decade, scientists have been researching the anticancer benefits of natural compounds since they can be utilized to prevent cancer with no discernible side effects.^[^ ^29^, ^30^, ^31^ ^]^ In our study, the methanolic extract of the flower showed notable anticancer activity against A549 cell lines, and the effect of *Chrysanthemum extract on various cancer cell lines (*liver, breast, and colon cancer cell lines) was reported previously.^[^ ^32^ ^]^ In our study, the anticancer effect was lower at low concentrations, and it improved at higher extract concentrations. The concentration‐dependent activity of *Chrysanthemum on* MCF‐7 cell lines was reported. Murayama et al. (2013) reported anticancer activity of *Chrysanthemum on* MCF‐7 cell lines, and maximum activity was achieved at higher concentrations (>25 μg mL^−1^).^[^ ^33^ ^]^ In Korean *Chrysanthemum* sp., the methanol extract decreased MCF‐7 cell viability. At a 200 μg mL^−1^ extract concentration, >45% cell suppression effect was observed.^[^ ^34^ ^]^ A recent study found that the aerial part of *Maresia nana* extract exhibited 58.50 ± 3.5% cytotoxicity in A549 cells at the maximal dose. Additionally, it also boosted HEK293 cell growth, suggesting that it was selectively cytotoxic to cancer cells.^[^ ^17^ ^]^

Apoptosis, also known as programmed cell death, has been linked to a variety of diseases, including cancer. The three basic processes that lead to apoptosis are intrinsic (mitochondrial mediated), extrinsic (death receptor mediated), and endoplasmic reticulum stress–dependent signaling transduction. Caspase‐8 is an important extrinsic pathway mediator; when activated, caspase‐8 induces apoptosis by activating the executioner caspases‐3, ‐6, and ‐7. Caspase‐9 is a key upstream mediator in the intrinsic pathway that triggers apoptosis by activating caspases‐3 and ‐7.^[^ ^35^ ^]^ Caspase‐3 exhibits both exogenous and endogenous apoptotic characteristics via interactions with caspase‐8 and ‐9.^[^ ^36^ ^]^ Furthermore, our qPCR results showed that *C. indicum* flowers and leaves extract increased the expression of *caspase‐*3, ‐8, and ‐9 mRNA levels while significantly reducing the mRNA levels of antiapoptotic genes *Bcl‐2* and *Bcl‐xL*, which is also accompanied by a rise in apoptosis‐promoting genes (*Bax*). *P* glycoprotein is a well‐established membrane transporter that may remove drug molecules from cancer cells, hence reducing the effectiveness of cancer therapy. Cancer cells upregulate the expression of *P*‐glycoprotein as a defensive mechanism to evade cell death caused by chemotherapy. The molecular investigation showed that *C. indicum* prevented the increase in both matrix metalloproteinase (MMP)‐3 and MMP‐9 while reducing the levels of tissue inhibitor of metalloproteinase 1 (TIMP‐1) in MRC‐5 fibroblast cells and A549 lung cancer cells.^[^ ^37^ ^]^ MMP‐induced cell apoptosis by targeting apoptotic proteins of Bax‐2, Bcl‐2, and cleaved caspase‐3.^[^ ^38^ ^]^ In addition, *C. indicum* promoted cell apoptosis by mediating the activities of caspases‐3/‐8 and upregulated the protein expression of *p53* and *miR‐29b* gene activation.^[^ ^39^ ^]^

Traditionally, indicators of apoptosis have been assessed using serological‐based methods such as enzyme‐linked immunosorbent assay (ELISA), Luminex, and cytometric bead array.^[^ ^40^ ^]^ However, apoptosis mRNA quantification is often used to study apoptosis mRNA using molecular‐based methods, especially in small samples. Compared with serological‐based methods, RT‐qPCR is the preferred technique for quickly and reliably measuring apoptotic indicators in cells, tissues, or tissue biopsies. RT‐qPCR offers more flexibility in this respect.^[^ ^41^, ^42^, ^43^, ^44^ ^]^

Antimicrobial resistance (AMR) is a serious public health concern that affects people all over the world by increasing the number of harmful organisms, including bacteria and fungi, that are resistant to one or more antimicrobial medications and are referred to as multiresistant organisms. Antibiotic resistance can occur through gene mutations, the production of antibiotic–hydrolytic enzymes, or the acquisition of resistance‐conferring genes through horizontal gene transfer, which occurs between bacteria of the same species and is thought to be the most important factor in today's AMR pandemic.^[^ ^45^ ^]^ Despite the enormous efforts made over the past decade, several antibacterial and anticancer natural plant‐derived products have been an invaluable and useful source of *anticancer* and antibacterial agents *over* the years.^[^ ^46^ ^]^ Globally, up to 200 species are considered medicinal plants, and about 25% to 50% of current pharmaceuticals are derived from plants.^[^ ^47^ ^]^ In this study, the methanol extract of flowers and leaves was tested for antibacterial activity. The flower extract showed maximum antibacterial activity on tested bacteria with MICs ranging from 7.81 ± 1.53 to 31.25 ± 2.45 μg mL^−1^, and this value was high in leaves (15.62 ± 3.22–125 ± 3.28 μg mL^−1^). *S. epidermidis* was highly susceptible to flowers and leaves extract of *C. indicum* than other bacteria. The degree of antimicrobial activity varied based on the presence of phytochemicals and solvent polarity. Shunying et al. (2005) reported the antibacterial activity of *C. indicum* aqueous extract against bacteria such as *Shigella flexneri*, *Streptococcus pneumoniae*, *Escherichia coli*, *Pseudomonas aeruginosa,* and *Staphylococcus aureus*.^[^ ^48^ ^]^ Shao et al. (2020) extracted *C. indicum* flower using ethanol, and it showed antibacterial activity against *B. subtilis* (258.75 mg mL^−1^).^[^ ^49^ ^]^ The Korean wild *Chrysanthemum* flowers have antibacterial properties against *S. aureus*, *P. aeruginosa*, and *Candida albicans*, as shown by the extracts obtained from these flowers.^[^ ^50^ ^]^ *C. indicum* showed efficacy against foodborne pathogens, including Gram‐positive and Gram‐negative bacteria such as *Salmonella typhi*, *Clostridium perfringens*, and *Listeria monocytogenes*.^[^ ^8^ ^]^ The effective extraction of polyphenolic chemicals, such as flavonoids, indicates their crucial role in producing antibacterial activity. The antibacterial activity or inhibition is induced by the reactions between the plant‐derived molecule and enzymes and proteins of the microbial cell membrane.^[^ ^51^ ^]^

We accept the limits and inadequacies of the approach used in this research. Further research is required to compare different fractions from the samples, as well as the phytochemical composition and biological activity of the fractions. Dual‐capillary polar and polar columns must be studied for polar and nonpolar volatile chemicals. To validate the recently found compounds, retention indices of the components need to be calculated using linear interpolation concerning the retention durations of two common n‐alkane mixes C_8_–C_20_ and C_21_–C_40_). An additional recognized limitation is the absence of tests on healthy cell lines, which would offer a more comprehensive understanding of the cytotoxic effects of the compounds being studied. Further studies are also required to identify the main components of methanolic extracts using high‐performance liquid chromatography–MS. The lack of in vivo research is one of the study's limitations. When assessing the possible biological effects of plant extracts against pathogenic bacteria and their antiproliferative activity against cell lines, in vivo research is essential for completing the picture left by in vitro data. Regarding target organ delivery, efficacy, and possible side effects, the in vivo setting is a great deal more complicated. To determine the effectiveness and safety of these substances, in vivo studies should be given priority in future research.

In conclusion, there are several bioactive compounds with notable antioxidant, anticancer, and antibacterial qualities present in the methanol extract of *C. indicum* flower and leaf. The methanolic extract of *C. indicum* flower and leaf extract contains unique bioactive terpenoids, which are unique to this study. It also shows significant antioxidant activity at low doses, potential anticancer effects at lower concentrations, and antimicrobial activities against Gram‐positive and Gram‐negative bacteria. These findings differ from those of previous reports. These results provide a strong basis for further research into its potential therapeutic uses as well as opportunities for the creation of natural pharmaceutical products. To evaluate their therapeutic potential, future research should concentrate on identifying and defining the particular bioactive compounds that produce these effects, as well as carrying out in vivo and clinical trials.

## 4 Experimental Section

4.1

4.1.1

##### C. indicum Flowers and Leaves Extraction

Commercial collections of dried flower heads and leaves of *C. indicum* were made in Riyadh, Saudi Arabia, in August 2023. The selected plant species was validated by Prof. Dr. Mohammed Fasil, and voucher specimens were deposited in the herbarium of the Department of Botany and Microbiology, College of Science, King Saud University, Riyadh, Saudi Arabia (KSU NO‐18811). The flowers and leaves of *C. indicum* were extracted with methanol as described previously by Lee et al.^[^ ^52^ ^]^ The fine powder of flowers (50 g) or leaves (50 g) was extracted with methanol (500 mL) and placed on a rotary shaker (100 rpm) for 24 h at 24 ± 1 °C. The extracted flowers and leaves were evaporated at a temperature (32 ± 1 °C) and maintained in an oven (60–80 °C) until constant weight was achieved.^[^ ^53^, ^54^ ^]^ The extract was stored at 4 °C for phytochemical studies and activity analysis.

##### Determination of Phytochemicals from the Methanol Extract

The sample volatiles were analyzed with an Agilent GC–MS 7890B GC system from Agilent Technologies (Santa Clara, CA, USA) as reported previously by Yang et al.^[^ ^55^ ^]^ A GC–MS instrument attached to an autosampler injection system received 1 μL of the sample after the extracts of leaves and flowers were reconstituted with acetone. Using a Durabond‐5‐5 MS capillary column (30 m length, 0.25 mm internal diameter, and 0.25 μm thickness), phytochemicals were found. A helium flow rate of 1 mL min^−1^ was used to elute the chemicals. The National Institute of Standards and Technology (NIST) and the Wiley database‐integrated software were used to identify the products. The parts with a matching factor of more than 90% were detected by comparing the components with those located in the computer libraries (Wiley and NIST) that were linked to the GC–MS apparatus.

##### Analysis of TPC and TFC

The TPC of the extract was analyzed as reported previously by Wolfe and Liu.^[^ ^56^ ^]^ Gallic acid was prepared from 20–200 μg mL^−1^ and used for the preparation of a standard curve. Briefly, 0.1 mL of each plant extract (1 mg mL^−1^) was combined with 2 mL of 20% NaHCO_3_, 0.5 mL of Folin–Ciocalteu reagent, and 3 mL of distilled water. The mixture was properly mixed and then incubated for 20 min at 45 °C. The optical density (OD) of the samples was measured using a spectrophotometer (U2001 UV–vis Spectrophotometer, Hitachi, Japan) at 725 nm. The linearity of the curve was assessed using the calibration curves’ coefficient of determination (R^2^). The amount of TPC in the sample was expressed as mg GAE g^−1^ of extract.

The amount of TFC was estimated as described previously by using the method reported by Ordonez et al.^[^ ^57^ ^]^ In summary, quercetin was prepared at various concentrations in Millipore water (20–200 μg mL^−1^), and the amount of QE was determined from the calibration curve. Then, 1 mL of AlCl_3_ that had been dissolved in ethanol was mixed with 500 μL of each extract. The mixture was then mixed with 3 mL of sodium acetate solution (50 g L^−1^). Following that, two acetic acid drops were added. The resultant solution was incubated at 23 °C for 30 min in a dark atmosphere. At a wavelength of 415 nm, the OD was determined using a spectrophotometer (U2001 UV–vis Spectrophotometer, Hitachi, Japan). The TPC was calculated using a standard curve that was developed from the QE standard. The results were expressed as mg QE g^−1^ of extract.

##### DPPH Scavenging Activity

The DPPH radicals scavenging assay was conducted utilizing the methodology outlined by Tian et al.^[^ ^58^ ^]^ *C. indicum* flower and leaf extracts at concentrations of 100, 200, and 400 μg mL^−1^ were prepared in 200 μL DPPH solution (2 mL, 0.08 mM) and 200 μL methanol solution. The reaction mixture was then left to incubate in total darkness for 30 min. A U2001 UV–vis Spectrophotometer (U2001 UV–vis Spectrophotometer, Hitachi, Japan) was used to detect the OD at 517 nm. Ascorbic acid (200 μL, 100–400 μg mL^−1^) served as the positive control. The OD was analyzed. Half‐maximal inhibitory concentration (IC_50_) values were calculated using Graph Pad Prism software (version 5.0, La Jolla, CA, USA), and the line equation was obtained from the percentage value of the antioxidant activity and the concentration value plotted on a graph, with the concentration value on the X axis and the percentage activity on the Y axis as described previously.^[^ ^59^ ^]^

##### ABTS Activity

The flowers and leaf extracts of *C. indicum* were used for the determination of ABTS free radical scavenging activity.^[^ ^60^ ^]^ The test was initially conducted using a mixture of the potassium persulphate solution (140 mM) and the ABTS solution (192 mg/50 mL). Consequently, the reaction mixture was allowed to sit at room temperature (25 °C) for ≈12 h in the dark. Additionally, the ABTS solution was mixed with methanol to achieve an absorbance of 0.70 ± 0.02 at 734 nm. Furthermore, 50 μL of each plant extract concentration was thoroughly mixed with 3 mL of diluted ABTS. The mixture was also incubated for 6 min in a dark atmosphere. Vitamin C (100–4000 μg mL^−1^) served as the positive control. The OD at 734 nm was then measured using a spectrophotometer (U2001 UV–vis Spectrophotometer, Hitachi, Japan). Results were expressed as ABTS% inhibition and IC_50_ values.^[^ ^61^ ^]^

##### Cytotoxicity Analysis

3‐(4,5‐dimethylthiazol‐2‐yl)‐2,5‐diphenyltetrazolium bromide (MTT) assay was performed for the determination of cytotoxicity. The human lung adenocarcinoma cell line A549 (ATCC:CCL‐185) was cultured in Dulbecco's modified Eagle medium with 1% penicillin–streptomycin and 10% fetal calf serum. A549 was seeded into 400 μL culture medium in 96‐well tissue culture plates. Then it was incubated at 37 °C for 24 h in a CO_2_ incubator, and the fresh culture medium was replaced after 24 h. The methanol extract of the leaves and flowers was diluted appropriately, and the final concentration was maintained between 50 and 400 μg mL^−1^ with cisplatin (30 μg mL^−1^) as the positive control and incubated at 37 °C for 24 h. To the microtiter plate, the MTT reagent (20 μL) was inoculated and further maintained for 2 h. To the wells, 100 μL of DMSO was added and incubated for 10 min. The color intensity of the sample was analyzed using a spectrophotometer at 570 nm. The viability of the A549 cell (%) was determined by three independent experiments. The mean value was considered for data processing, and the IC_50_ value was subsequently determined using the GraphPad Prism software (version 5.0, La Jolla, CA, USA).

##### Gene Expression Analysis

Gene expression of apoptotic (*caspase‐3*, −*8*, −*9*, *Bax*) and antiapoptotic (*Bcl‐xL*, *Bcl‐2*) markers was determined via RT‐qPCR using RNA extracted from A549 cells treated with plant extracts. Precisely 2 × 10^5^ A549 was seeded into six‐well plates containing 3 mL of culture medium. The culture medium was then mixed with flowers and leaves of *C. indicum* (100 μg mL^−1^). After 24 h, the A549 cell was digested with trypsin (0.02%) and was further centrifuged using a refrigerated centrifuge (10 000 × g) for 10 min. After 10 min, the pellet was reconstituted with PCR buffer and used for RT‐qPCR analysis. To determine the apoptosis coding genes, RNA extraction was performed using the RNeasy kit as described by the instructions given by the manufacturer (Qiagen, Hilden, Germany). The RT‐qPCR reaction was carried out in a 7500 Fast real‐time PCR (7500 Fast; Applied Biosystems, USA) using the RT‐qPCR methodology as described in our recent study.^[^ ^62^ ^]^

##### Screening for Antibacterial Activity

Antibacterial activity of the methanol extract was performed using the following bacterial pathogens (Gram positive and Gram negative). The selected Gram‐positive strains were *Staphylococcus aureus* (MTCC‐29213), *Staphylococcus epidermidis* (MTCC‐12228), and *Bacillus subtilis* (MTCC‐10400). A total of three Gram‐negative strains, *Escherichia coli* (ATCC‐25922), *Klebsiella pneumoniae* (MTCC‐13883), and *Pseudomonas aeruginosa* (MTCC‐27853), were used. All these bacterial strains were obtained from the King Khalid University Hospital, Riyadh, Saudi Arabia.

##### Disc Diffusion Method

The agar disc diffusion method is considered the gold standard method and was used in this study as described previously,^[^ ^63^ ^]^ with minor modifications. Subculture from the 6 bacterial spp. was prepared in 100 μL at 1.0 × 10^7^ CFU mL^−1^ on Muller–Hinton agar (MHA). About 0.1 mL of bacterial inoculum was spread on MHA plates using an “L” rod. To create wells, holes with 5 mm diameters were punched at equal intervals using a sterilized cork borer. Next, different concentrations of flowers and leaves of *C. indicum* (125, 250, 500, and 1,000 μg mL^−1^; 100 μl well^−1^) were added to the wells and incubated for 24 h 37 °C to promote microbial growth and assess the zone of inhibition around each well, and the antibacterial activity was measured.^[^ ^64^ ^]^

##### Analysis of MIC and MBC

The MIC and MBC were tested using the broth dilution method as described earlier by Basri and Sandra^[^ ^65^ ^]^ using the 2,3,5‐triphenyl tetrazolium chloride (TTC). Initially, a primary culture of pathogenic bacteria was established at a 5 × 10^6^ CFU mL^−1^ concentration. MHB was then used to dilute 1.95–1000 μg mL^−1^ of the extract. In a 96‐well plate, 100 μL of the extract was added to each well before bacteria were added. The plate was incubated at 37 °C for a whole day. The emergence of a red color indicated the proliferation of microorganisms in each well after the addition of 20 μL (2 mg mL^−1^) of TTC. At a concentration referred to as the minimum inhibitory concentration (MIC), no color shift was observed. Each well's 100 μL of contents that did not change color was cultivated on MHA and kept at 37 °C for a whole day. MBC was the lowest dilution at which growth was inhibited.^[^ ^66^ ^]^

##### Statistical Analysis

GraphPad Prism (version 5.0, La Jolla, CA, USA) software was used to analyze the obtained results. The results were expressed as mean  ±  standard deviation, and statistical analysis was performed using Analysis of Variance. The Student's unpaired *t*‐test was used to compare the means of two independent groups. For non‐Gaussian variables, the Mann–Whitney *U* test was used to compare the groups. Differences were considered significant when the *P* value was <0.05.

## Conflict of Interest

The authors declare no conflict of interest.

## Author Contributions

**Tarad Abalkhail**: investigation (equal); resources (equal); and writing—original draft (equal). **Noorah A. Alkubaisi**: funding acquisition (lead); investigation (equal); resources: (equal); and writing—original draft (equal). **Ibrahim M. Aziz**: data curation (lead); project administration: (lead); validation (lead); and writing—original draft (equal). **Mashail Fahad Alsayed**: formal analysis (lead) and writing—review and editing (equal). **Hissah Abdulrahman Alodaini**: data curation (lead) and writing—review a editing (equal). **Amal Saad AL‐Shenifi**: writing—review and editing (equal). **Mohamed A. Farrag**: investigation (lead) and writing—review and editing (supporting).

## Data Availability

The data that support the findings of this study are available from the corresponding author upon reasonable request.
